# Stage Progression and Neurological Symptoms in *Trypanosoma brucei rhodesiense* Sleeping Sickness: Role of the CNS Inflammatory Response

**DOI:** 10.1371/journal.pntd.0001857

**Published:** 2012-10-25

**Authors:** Lorna MacLean, Hansotto Reiber, Peter G. E. Kennedy, Jeremy M. Sternberg

**Affiliations:** 1 Centre for Immunology and Infection, Department of Biology, Hull York Medical School, University of York, York, United Kingdom; 2 CSF and Complexity Studies, Berlin, Germany; 3 Institute of Infection, Immunity and Inflammation, College of Veterinary and Life Sciences, University of Glasgow, Glasgow, United Kingdom; 4 Institute of Biological and Environmental Sciences, University of Aberdeen, Aberdeen, United Kingdom; Foundation for Innovative New Diagnostics (FIND), Switzerland

## Abstract

**Background:**

Human African trypanosomiasis progresses from an early (hemolymphatic) stage, through CNS invasion to the late (meningoencephalitic) stage. In experimental infections disease progression is associated with neuroinflammatory responses and neurological symptoms, but this concept requires evaluation in African trypanosomiasis patients, where correct diagnosis of the disease stage is of critical therapeutic importance.

**Methodology/Principal Findings:**

This was a retrospective study on a cohort of 115 *T.b.rhodesiense* HAT patients recruited in Eastern Uganda. Paired plasma and CSF samples allowed the measurement of peripheral and CNS immunoglobulin and of CSF cytokine synthesis. Cytokine and immunoglobulin expression were evaluated in relation to disease duration, stage progression and neurological symptoms. Neurological symptoms were not related to stage progression (with the exception of moderate coma). Increases in CNS immunoglobulin, IL-10 and TNF-α synthesis were associated with stage progression and were mirrored by a reduction in TGF-β levels in the CSF. There were no significant associations between CNS immunoglobulin and cytokine production and neurological signs of disease with the exception of moderate coma cases. Within the study group we identified diagnostically early stage cases with no CSF pleocytosis but intrathecal immunoglobulin synthesis and diagnostically late stage cases with marginal CSF pleocytosis and no detectable trypanosomes in the CSF.

**Conclusions:**

Our results demonstrate that there is not a direct linkage between stage progression, neurological signs of infection and neuroinflammatory responses in *rhodesiense* HAT. Neurological signs are observed in both early and late stages, and while intrathecal immunoglobulin synthesis is associated with neurological signs, these are also observed in cases lacking a CNS inflammatory response. While there is an increase in inflammatory cytokine production with stage progression, this is paralleled by increases in CSF IL-10. As stage diagnostics, the CSF immunoglobulins and cytokines studied do not have sufficient sensitivity to be of clinical value.

## Author Summary

Human African trypanosomiasis, caused by the parasites *Trypanosoma brucei rhodesiense* and *T.b.gambiense*, is clinically defined by two diagnostic stages, an early stage where the parasites appear to be localised to the blood and lymphatic systems, and a late stage where the parasites are also localised in the central nervous system (CNS) and cause a meningoencephalitis, which is fatal if untreated. We studied the progression between these stages of infection in *T.b.rhodesiense* infections in Uganda. Progression from early to late stage was associated with an increase in inflammatory responses in the CNS as measured by analysis of the cerebrospinal fluid. However, contrary to predictions based on experimental model studies, neither disease stage progression nor CNS inflammatory responses were directly associated with development of neurological symptoms. Our results suggest that biological basis of the boundary between the two diagnostic stages in this infection may not be clear cut, with implications for therapeutic decision making.

## Introduction

Human African trypanosomiasis (HAT), also known as Sleeping Sickness, is caused by the protozoan hemoflagellate *Trypanosoma brucei ssp*. After inoculation of the parasite by the tsetse fly vector, the disease progresses through two stages. In the hemolymphatic, or early stage of disease, parasites proliferate in the blood and lymphatic system. In the meningoencephalitic, or late stage, parasites penetrate the blood brain barrier (BBB) and persist and proliferate in the CNS, causing an encephalitic reaction that leads to death if untreated or inadequately treated [1]. Two sub-species of African trypanosome give rise to HAT. *T.b.gambiense* is endemic to West and Central Africa, with a chronic course of infection in which late stage may not commence for months or years after infection, and for which there is recent evidence for asymptomatic infection [2], [3], [4]. *T.b.rhodesiense* is endemic in East and Southern Africa, is distinguished by the SRA (serum resistance associated gene) and exhibits a more acute pattern of progression than *T.b.gambiense*, although there is considerable diversity in progression rate that may be related to parasite virulence variation and host immunogenetics [5].

Animal model studies of *T.brucei* infection demonstrate that dysregulated inflammatory responses are a major contributor to the pathophysiology of infection, both systemically [6] and in the brain [7], [8], where it was hypothesised that the development of neuropathology is associated with an astrocytosis regulated by the CNS inflammatory/counter-inflammatory cytokine balance [9].

In humans, direct measurements of immune cell activation in the brain are not possible for obvious ethical reasons. While gross inflammatory pathology analogous to that observed in rodent models has been described in *post-mortem* material [10], our limited understanding of the pathophysiology of CNS infection in HAT derives from the observation of neurological symptoms and analysis of patients' cerebrospinal fluid (CSF) [11], [12], [13]. A spectrum of neurological symptoms is observed in HAT infection. This includes sleep, sensory, motor and psychiatric disorders as well as the characteristic sleep disturbances that have given this disease its common name of Sleeping Sickness [1].

Staging is critical to therapeutic decision making as late stage infections of *T.b.rhodesiense* are currently treated with arsenical drugs that induce a severe and sometimes fatal reaction known as the post-treatment reactive encephalopathy (PTRE) in about 10% of treated patients, half of whom die as a result giving an overall drug mortality of 5% [12]. Currently, disease staging primarily relies on the detection of trypanosomes in the CSF and/or an elevation in the CSF white blood cell (WBC) count. The most widely applied diagnostic cut off for CSF WBC counts to indicate a late stage infection (WHO criteria) is >5 cells/µl [14], although in the case of *T.b.gambiense* infection there is some evidence that this value is too low and that effective early stage treatment may still be administered in patients with up to 20 WBC/µl in the CSF. It has also been proposed that HAT cases with between 5 and 20 CSF WBC/µl fall into an intermediate stage category, regardless of whether trypanosomes are detected in the CSF [11]. In addition to CSF cell counts, other biochemical and immunological markers have been investigated to improve the sensitivity and specificity of diagnostic staging. In late stage HAT there is an increase in CSF protein level that is largely accounted for by immunoglobulin (Ig) expression. High levels of intrathecal IgM synthesis are typical and have been shown to be a sensitive marker for intrathecal inflammatory responses and therefore of stage diagnostic value in *T.b.gambiense* infections [15]. A number of stage-specific alterations in cytokine and chemokine response have also been described in CSF from HAT patients [8], [16], [17], [18], and IL10 has been proposed as a potential diagnostic marker for infection and cure owing to the speed with which levels return to normal after treatment [19].

As part of a study of the clinical evolution of *T.b.rhodesiense* HAT [20], we have analysed in detail the parasitological and clinical progression of HAT in a cohort of patients recruited in Serere in 2003, in Eastern Uganda. Because the parasites circulating in this epidemic were genetically homogeneous [21], and the host population comprised a single ethnolinguistic group, this set of cases offers an opportunity to describe the evolution of HAT independent of variations in parasite virulence, and to use the clinical data to explore the relationship of inflammatory responses in the CNS to clinical disease. In particular, we tested the hypothesis that disease progression and neurological dysfunction would be associated with increasing inflammatory (agonist) and decreasing anti-inflammatory (antagonist) responses. We also evaluated the stage diagnostic potential of CSF immunoglobulin and cytokine responses in *T.b.rhodesiense* infection.

## Methods

### Ethics statement

This study was conducted according to the principles expressed in the Declaration of Helsinki. All patients recruited received written and verbal information explaining the purpose of this study and gave informed written consent. All protocols were approved by ethics committees in Uganda (Ministry of Health) and UK (Grampian Joint Ethics Committee). Ethical consent forms were designed in English and also translated into local languages. Consent was given as a signature or a thumb-print after verbal explanation. For those under 16 consent was given by their legal guardian, and for those whose clinical condition prohibited full understanding of the recruitment process, consent was gained from a spouse or other family member.

### Patient study sites and recruitment

115 HAT patients were recruited at Serere Health Centre, Serere District, Eastern Uganda, between August 2002 and July 2003. This cohort of cases was drawn from of a larger multi-centre study, for which study sites, recruitment protocols, treatment regimens, disease progression characteristics and clinical examination methods have been published elsewhere [20]. All patients belonged to the Ateso (Eastern Nilosaharan) ethnolinguistic group. Patients with intercurrent infections of malaria, filariasis or schistosomiasis were excluded from the study.

### Staging criteria

Staging was carried out in accordance with WHO criteria [14]. These define late stage by the presence of parasites in the lumbar CSF and/or a CSF WBC>5/µl in parasitemic individuals. In this cohort, parasite counts in the CSF were determined by Neubauer hemocytometer, and the definitive presence or absence of parasites in the CSF by double centrifugation [22].

### CSF and plasma analysis

Plasma and lumbar CSF were collected from all patients as part of routine diagnostic and stage determination procedures. Paired plasma and CSF samples were frozen in liquid nitrogen within 1 h of collection, and maintained in liquid nitrogen until required for analysis.

Control plasma and CSF samples for cytokine analysis were obtained from 17 HAT suspects at Serere Health Centre and 18 HAT suspects at the LIRI Health Centre, Tororo, Uganda respectively who were all later diagnosed as non-infected. In some assays, limitations on the volume of plasma and CSF available meant that a subset of patient samples that was effectively randomly selected was analysed. Cytokines IFN-γ, IL-6 and IL-10 concentrations were measured in CSF using a solid phase analyte capture sandwich ELISA (OptiEIA set, Becton Dickinson-Pharmingen, Oxford, U.K.) as previously described in [23]. Free TNF-α was measured using a receptor binding assay as described previously (BioLISA, Bender Med Systems, Wien, Austria) [24]. Cytokine assays limits of detection were IFN-γ:1.8 pg/ml; IL-10:1.6 pg/ml; TGF-β:19.2 pg/ml; IL-6:8.3 pg/ml; TNF-α:22 pg/ml. For descriptive and inferential statistical analysis, results below the limit of detection were assumed to be (0.5× limit of detection value). As rank statistical methods were used this assumption did not bias significance tests. Total IgM, IgA, IgG and albumin were determined by nephelometry (ProSpec, Dade-Behring, Marburg, Germany) as described in [15].

### Blood-CSF barrier and intrathecal humoral responses

Blood-CSF barrier function was evaluated using the albumin quotient (*Q*_ALB_). The cut off for dysfunction was calculated using the formula
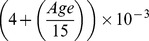
Intrathecal immunoglobulin synthesis (Ig_LOC_) was evaluated using the method of Reiber [25]. Briefly, this analytic approach is based on a reference set of 4300 normal CSF samples, from which an upper hyperbolic discrimination curve *Q*_LIM_ defines the upper limit of the immunoglobulin quotient (*Q*(Ig)) in the absence of intrathecal Ig synthesis. Intrathecal Ig synthesis results in a *Q*(Ig) lying above *Q*_LIM_. The level of intrathecal synthesis of each isotype (Ig_LOC_) is derived from the formula
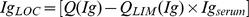
The results of this analysis were presented in quotient diagrams [26] using CSF Statistics Tool software (CoMed GmbH, Soest, Germany).

### Data analysis and statistics

None of the continuous variable parameters examined could be transformed to normality. Therefore differences between groups were tested using the Mann-Whitney U-test, or the Kruskal Wallis test followed by Dunn's post-hoc test. Bivariate correlations were evaluated using Spearmann's rank correlation coefficient (r_s_). Diagnostic outcomes as dependent variables were tested on cytokine concentrations using logistic regression, and diagnostic power was assessed using receiver operating characteristic analysis. Diagnostic panel candidates were selected using mixed stepwise logistic regression and optimal diagnostic cut offs were determined using the prediction profiler in JMP6.0 (SAS Institute, Cary, NC, USA).

## Results

### Study population

The study population comprised 35 early stage and 80 late stage patients. The age, gender and diagnostic stage of these individuals are presented in Table 1. There were no significant associations between either gender or age and infection stage. Of the 80 late stage cases, 77 were confirmed by detection of trypanosomes in the CSF after double centrifugation. The remaining 3 where trypanosomes could not be detected exhibited WBC counts between 6 and 20/µl. Progression to late stage in this focus was rapid with a median reported duration of illness of 8 weeks for late stage cases.

**Table 1:** Characteristics of study population.

Diagnostic stage	Early	Late
Population(n)	35	80
Male	16	32
Female	19	48
Age (median, range)	20 (2–68)	21 (3–85)
Reported duration of infection, days (median, range)	25 (2–90)	57 (6–157)

### Parasitaemia and CSF cell counts

Bloodstream parasitemias were scored on thick blood films. Late stage median parasitemia was lower than in early stage, but this difference was not significant (Table 2). However, parasitemia was significantly inversely correlated to reported duration of illness. In contrast, both CSF trypanosome and WBC counts increased significantly with increasing duration of illness (r_s_ = 0.53 p<0.0001 and 0.42 p<0.0001 respectively). Also, as would be expected given that CSF trypanosome and WBC are diagnostic criteria, both were significantly higher in late stage cases compared to early stage cases.

**Table 2:** Serum and CSF albumin and immunoglobulin concentrations by stage of infection.

Parameter	Reference range	Early^a^	Late^a^	Correlation with duration of illness^b^
**Serum**				
IgG (g/L)	8.0–18.0	20.9 (16.6–25.3)	22.5 (18.9–27.5)	0.18
IgA (g/L)	0.9–45.0	1.9 (1.3–2.7)	2.1 (1.6–2.6)	0.09
IgM (g/L)	0.6–2.5	6.3 (2.0–14.9)	13.6 (9.0–23.3)***	0.35***
Albumin (g/L)	35.0–55.0	24.9 (21.1–31.6)	22.0 (17.6–26.9)*	−0.28**
**CSF**				
IgG (mg/L)	<40.0	27.6 (21.4–38.1)	76.0 (45.1–154)***	0.39**
IgA (mg/L)	<6.0	1.1 (0.7–1.6)	6.0 (2.3–13.4)***	0.46**
IgM (mg/L)	<1.0	0.66 (0.29–2.7)	64.2 (22.6–178)***	0.6***
Albumin (mg/L)	<350	72.0 (46.5–102)	102 (72.5–190)***	0.1
Protein (mg/L)	<500	171 (133–238)	365 (230–644)***	0.35***
White cells/µl	<5	3 (2–5)	34 (12–76)***	0.43***
Parasitemia^c^		3.5 (2–16.7)	2 (0.5–4)	−0.2*
CSF trypanosomes/ml		0.0 (0.0–0.0)	2.5 (1.5–6.0)***	0.53***
Q_ALB_		2.7 (2.0–3.6)	5.0 (3.2–7.7)***	0.27**

a : Data are median (interquartile range). Difference between early and late stage parameters tested for significance using Mann-Whitney U-test.

b : Spearmann rank correlation coefficient.

Significance levels indicated:

* : p<0.05.

** : p<0.01.

*** : p<0.0001.

c : Parasitaemia – thick blood film parasites per 10 fields at ×400 magnification.

### Plasma and CSF protein and albumin

Total CSF protein was significantly higher in late stage cases compared to early stage cases and also increased in relation to duration of disease (Table 2). Plasma albumin concentration was below the normal reference range for European populations [15] in both early and late stage cases and decreased with disease progression as measured by both disease stage and reported duration of illness (Table 2). In contrast, CSF albumin concentration was significantly higher in late stage cases compared to early stage cases. Likewise, the albumin quotient was significantly increased in late stage cases compared to early stage cases and also correlated to reported duration of disease (Table 2). Using the age related cut off for normal albumin quotient (Q_ALB_), blood brain barrier (BBB) dysfunction was indicated in 6% of early stage cases and 42% of late stage cases.

### Plasma and intrathecal immunoglobulin responses

Plasma IgG and IgA levels (Table 2) did not differ significantly between early and late stage cases and fell within the normal European reference range [15]. However plasma IgM levels in both early and late stage cases were increased above the reference range and increased with disease progression as estimated by stage of infection and duration of disease. In early stage CSF samples, all Ig isotype concentrations were within the reference range. However, in late stage CSF samples IgM, IgG and IgA concentrations were significantly increased compared to both early stage and normal reference range values, and also were significantly correlated with duration of disease. Intrathecal synthesis of immunglobulins was determined (mg/l) in relation to the upper hyperbolic discrimination line (Q_Lim_) [26] in quotient diagrams (Figure S1). Intrathecal Ig synthesis was detected in 12% early and 74% late stage cases (Table 3). The proportion of intrathecally synthesised immunoglobulin in relation to total CSF concentration also varied according to isotype, reaching a median of 44% in the case of IgM synthesis in late stage cases. Overall, of those late stage cases (n = 54) where intrathecal IgM synthesis was detected, intrathecal synthesis of a second isotype occurred in 61% (IgA) and 35% (IgG) of cases respectively. Intrathecal synthesis of all 3 isotypes was detected in 30% of these cases. There was also a significant correlation of intrathecal immunoglobulin synthesis with duration of illness, CSF trypanosome concentration and CSF white cell concentration for all isotypes.

**Table 3:** Intrathecal immunoglobulin synthesis by diagnosis stage and reported duration of infection.

	IgG	IgM	IgA	Any Intrathecal synthesis
**Early Stage n = 32**				
% Cases with intrathecal synthesis^a^	6	9	3	12
Local synthesis (Ig_LOC_) mg/l Median (IQR)	0.0 (0.0–0.0)	0.0 (0.0–0.0)	0.0 (0.0–0.0)	
**Late Stage n = 74**				
% Cases with Intrathecal synthesis	26	73***	46 ***	73 ***
Local synthesis (Ig_LOC_) mg/l Median (IQR)	0.0 (0.0–8.2)	44.5 (0.0–163.0)	0.0 (0.0–3.4)	
Correlation of local synthesis (Ig_LOC_) with disease duration (r_s_)	0.22 ¶	0.33 ¶¶	0.51 ¶¶¶	

a : intrathecal synthesis where *Q*(Ig)>*Q*_LIM_.

Significantly higher frequency in late stage cases compared to early stage cases * and *** p<0.05 and p<0.0001 respectively, fishers exact test.

Significant correlation of intrathecally synthesised Ig concentration with duration of illness. ¶, ¶¶ and ¶¶¶, p<0.05, p<0.01 and P<0.001 respectively, Spearman correlation test.

### CSF cytokine levels

**Figure 1:**
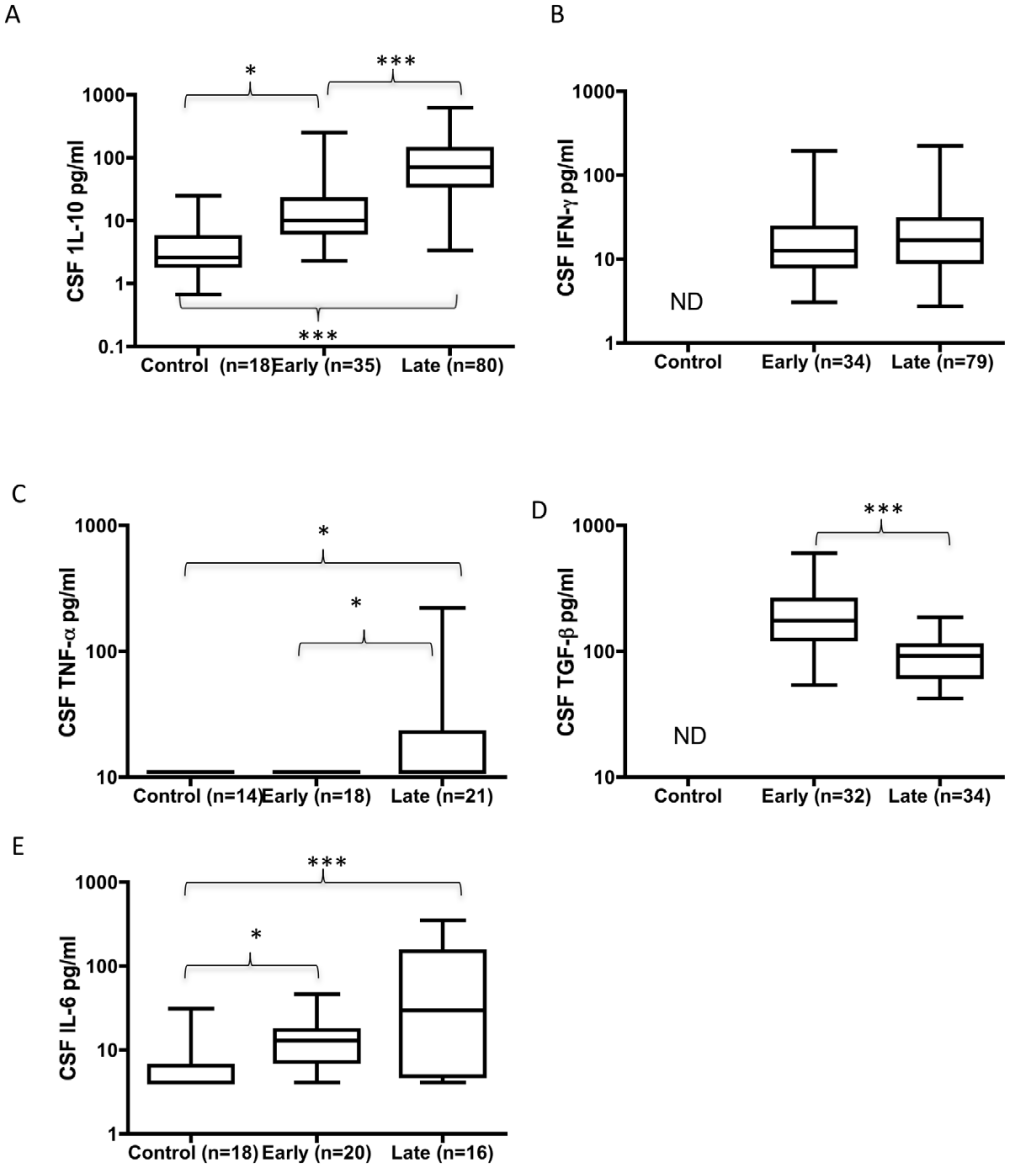
CSF cytokine concentrations in HAT. IL-10 (a), IFN-γ (b), TNF-α (c), TGF-β (d) and IL-6 (e) concentrations in control, early and late stage HAT patients. Boxes indicate median and interquartile range, and whiskers represent 10^th^ and 90^th^ percentiles. ***p<0.001; *p<0.05 Mann-Whitney U test. BLD = below limit of detection. ND: Not analysed due to insufficient material.

The CSF IL-10 (Figure 1a) concentration was significantly increased over control in both early and late infection stages and also increased with progression from early to late stage. The IL-10 concentration also showed a significant positive correlation with disease duration (r_s_ = 0.41 p<0.001). There was no significant difference between early and late stage cases for IFN-γ (Figure 1b). TNF-α levels (Figure 1c) in all control and early stage CSF samples were below the assay limit of detection, but were detectable at a significant level in 5/21 late stage cases. TGF-β levels (Figure 1d) were significantly higher in early stage samples compared to late stage although there was no significant correlation with duration of infection. The CSF IL-6 (Figure 1e) concentration was increased above control levels in both early and late stage cases, there was also significant correlation to duration of infection (r_s_ = 0.46 p<0.01).

When the relationships between the expression of each of the CSF cytokines were evaluated, significant positive relationships were identified between TNF-α and IL-10 (r_s_ = 0.42 p<0.01); IL-6 and IL-10 (r_s_- = 0.38 p<0.05); IL-6 and IFN-γ (r_s_ = 0.37 p<0.05) and a negative relationship between TGF-β and IL10 (r_s_ = −0.4 p<0.001).

IL-10 and TGF-β concentrations were significantly predictive of diagnosis stage in univariate logistic regression (likelihood ratio test p<0.0001 for both cytokines). Each log unit increase in CSF IL-10 and TGF-β concentrations were associated with odds ratios (OR [95% CI]) for late stage diagnosis of 4.0 (2.5–7.4) and 0.04 (0.01–0.0.17) respectively. To determine if either would be of utility as a late stage diagnostic, receiver operating characteristic (ROC) curves were analysed. For IL-10 the area under the ROC curve (AUROCC) was 0.85 but in order to achieve 100% specificity a cut off value of 275 pg/ml only offered 14% sensitivity. For TGF-β, the AUROCC was 0.86, and similarly the cut off for 100% specificity (50 pg/ml) only offered 5% sensitivity. An optimal combined panel of CSF IL-10, TGF-β, and IgM concentration was identified using stepwise logistic regression analysis and with discriminatory cut off levels for late stage determined on the patient data of IL-10>66.4 pg/ml; TGF-β<159.5 pg/ml and CSF IgM>89.2 mg/l. This panel offered an AUROCC of 0.97, with 70% sensitivity for 100% late stage specificity.

### Stage progression and neurological signs

Neurological signs including altered gait, tremors, incontinence, cranial nerve neuropathy (facial nerve palsies), somnolence and reduced Glasgow coma score (GCS) were observed in both early and late stage patients, indicating early onset of neurological involvement, with only moderate coma (GCS<12) being unrepresented in any early stage cases (Table 4). Of the neurological signs assessed, only somnolence was observed to show a significant (p<0.01) increase in frequency in relation to duration of illness (OR for each log unit increase in duration of illness = 2.1 (95% CI = 1.3–3.8)).

**Table 4:** Neurological signs in early and late stage HAT patients.

Neurological Sign	%Early	%Late
	n = 35	n = 80
Gait Ataxia	63	52
Tremors	60	69
Incontinence	20	15
Neuropathy	29	31
Somnolence	57	55
GCS<15	14	14
GCS<12	0	10*

* p<0.05 Mann-Whitney U test.

### CNS immune responses and neurological signs

The relationships of neurological signs to intrathecal immunoglobulin and cytokine synthesis were examined. There were no significant differences in either intrathecal immunoglobulin levels (Ig_loc_) or cytokine concentrations (IL-10, IFN-γ, TNF-α, TGF-β or IL-6) in relation to the presence or absence of gait ataxia, tremors, urinary incontinence, although cases with facial nerve palsies exhibited a slight increase in intrathecal IgA concentration (median (IQR) 0.0 (0.0–4.6) mg/l versus 0.0 (0.0–0.8) mg/l p<0.05). However, cases with moderate coma (GCS<13) exhibited substantially and significantly higher levels of intrathecal synthesis of all Ig isotypes as well as IL-10 and IL-6 (Table 5). This effect was not observed in cases with mild coma (GCS 13–14).

**Table 5:** Relationship of neurological impairment to intrathecal humoral response (Ig_LOC_) and cytokine responses.

CSF immune response	Glasgow Coma Score Categories		
	GCS = 15 (normal)	GCS 13–14 (mild coma)	GCS<13 (moderate coma)
	n = 91	n = 8	n = 8
IgG mg/l	0.0 (0.0–0.0)	0.0 (0.0–0.0)	70.5 (0.0–250.6)**
IgA mg/l	0.0 (0.0–1.1)	0.0 (0.0–1.0)	9.5 (4.4–11.8)***
IgM mg/l	0.5 (0.0–65.0)	20.1 (0.0–209.2)	178.0 (86.5–413.8)**
IL-10 pg/ml	40.9 (12.6–104.3)	27.1 (7.3–86.1)	266.9 (94.4–351.8)**
IL-6 pg/ml	12.0 (6.8–17.5)	17.8 (9.1–41.5)	172.7 (78.8–257.7)**

Note: All data are median (IQR). Significant difference to response in normal GCS cases (Dunns post hoc test) indicated in bold and.

** p<0.01.

*** p<0.001.

### HAT patients with CSF white cell counts between 5 and 20/µl

In *T.b.gambiense* infection, it has been proposed that CSF white cell counts of 5–20/µl, regardless of whether trypanosomes are detected in the CSF, should be regarded as an intermediate stage [11] that may be treated with pentamidine. In the cohort of *T.b.rhodesiense* patients described in this paper, only 3 individuals fell into this category. These patients were treated successfully with melarsoprol and followed up for two years. The characteristics of these subjects are presented in Table 6. All presented with normal GCS, gait, and an absence of somnolence. All exhibited normal BBB function. Unlike the late stage cases classified with white cell counts >20/µl that all were positive for CSF trypanosomes after double centrifugation, all three of these possible “intermediate” stage cases were negative for CSF trypanosomes. However 1/3 exhibited incontinence and cranial neuropathy. Furthermore, 2/3 exhibited intense intrathecal IgM synthesis, and 1/3 intrathecal IgA and IgG synthesis (Table 6 and Figure 1b closed triangles).

**Table 6:** Details of potential “intermediate-stage” cases.

Case	Sex	Age	GCS	CSF WBC/µl	CSF DC^a^	Normal gait?	Tremors?	Incontinence?	Neuropathy?	Somnolence?	Q_ALB_ Normal?	IgG IF%	IgA IF%	IgM IF%
Ser72	F	28	15	10	nil	Y	N	N	N	N	Y	0	0	85
Ser40	M	13	15	9	nil	Y	N	Y	Y	N	Y	0	0	0
Ser99	M	33	15	16	nil	Y	N	N	N	N	Y	35	35	90

a : CSF DC: Detection of trypanosomes after double centrifugation of the CSF.

## Discussion

Our understanding of pathogenesis in HAT is limited by the logistic difficulties of clinical studies in endemic areas. Studies in animal models suggest that the development of systemic and CNS pathology follows a dysregulation of host-inflammatory responses, and that host-immune response variation may control the severity of pathology [27]. African trypanosome CNS infection model studies indicate that the development of neuropathology results from inflammatory responses in the brain and it has been hypothesised that pathogenesis may be controlled or limited by counter-inflammatory responses [8].

In this study we selected a cohort of *T.b.rhodesiense* HAT patients from Eastern Uganda who were exposed to a genetically homogeneous parasite population [21] and were drawn from a homogeneous ethnolinguistic (Ateso) host population. This approach enables us to minimise any confounding effects of natural parasite variability [5] on the parameters and also to a limited extent the effects of host genetic diversity on disease progression assuming the close relationship between language and genetic variation observed in other nilo-saharan populations [28]. We analysed the development of CNS humoral and cellular immune responses and their relationship to disease progression and neurological signs of HAT. Disease progression was assessed both in relation to diagnostic staging and the reported duration of disease.

Development of disease in the study group reported here was rapid, with a median disease period of 8 weeks for late stage infection, consistent with results previously reported from a larger cohort of patients from the Soroti focus [20]. Overall parasitemia levels were highest in early stage cases and fell in relation to duration of infection.

In the plasma, IgM concentration increased with both progression from early to late stage and reported disease duration. No similar increases were evident for plasma IgA and plasma IgG, which also remained within or close to the reference range concentrations described for healthy European subjects. This result is consistent with observations in both mouse and bovine models of a predominant polyclonal B-cell activation in trypanosomiasis [29]. Although IgM responses have been previously described in *T.b.gambiense* HAT, there has only been one previous report of elevated IgM in the serum and CSF of *T.b.rhodesiense* HAT patients [30]. Plasma albumin levels fell with disease progression. While this phenomenon may be expected as part of a negative acute phase response in early infection [31], in more chronic infection it is possibly also a consequence of liver pathology, albuminuria resulting from kidney damage, and cachexia [32].

In the CSF, total protein increased with stage progression and reported disease duration, consistent with previous observations for both *T.b.gambiense* and *T.b.rhodesiense*. The CSF protein level has been recognised as a useful stage diagnostic tool in HAT [14] but it is interesting to note that in this study of *T.b.rhodesiense* patients, the median late stage CSF protein concentration is lower than the recommended diagnostic cut off for staging employed in *T.b.gambiense* cases (370 mg/l). The increase in CSF protein is accounted for at least in part by the large and significant increases in CSF IgM, IgG and IgA concentration. Similar increases have been described previously for *T.b.gambiense* infection [15]. The increase in CSF immunoglobulin level is a product of both intrathecal immunoglobulin synthesis and accumulation of serum-derived immunoglobulins in the CSF as a result of a reduction in CSF flow and turnover rate [25], [33]. This is evident in this study from the significant increase in Q_ALB_ levels with both stage progression and disease duration, and indicates a reduction of either CSF production in the choroid plexus or outflow of CSF into venous circulation. In order to measure intrathecal synthesis of immunoglobulin we used the hyperbolic discrimination curve Q_LIM_ as cut off for non-CNS derived Ig [33]. Intrathecal Ig synthesis (Ig_LOC_) was detected in few (12%) early stage cases but commonly (70%) in late stage cases. In contrast to the findings from similar studies in *T.b.gambiense* infection [15], intrathecal Ig synthesis in *T.b.rhodesiense* HAT was predominantly a single class (IgM) response and 2 or 3 class responses were considerably less common. Overall, while intrathecal Ig synthesis in late stage infection provides clear evidence of the activation of humoral immune responses by trypanosomes in the CNS, it does not offer the sensitivity or specificity required to be effective as a stage diagnostic. None of the early stage cases in this study where intrathecal Ig synthesis was detected relapsed (over a 1 year follow up period) after suramin treatment, indicating that the diagnostic decision to classify these cases as early stage was correct. In *T.b.gambiense* infection a similar frequency of early stage cases exhibiting Ig synthesis has been described [15].

CSF cytokine concentrations were measured as indicators of cellular immune activation in the CNS. Increases in IL-10 and IL-6 concentration with stage progression are consistent with previous studies in both *T.b.rhodesiense* [19] and *T.b.gambiense* infection [16]. In this study we further observed an increase in TNF-α concentration and a decrease in TGF-β concentration with progression to late stage. The increases in CSF cytokine concentrations were not restricted to late stage cases only. CSF concentrations of IL-6 and IL-10 were also elevated over control levels in early cases, and provide evidence of early activation of CNS cellular responses and suggest, as has been shown in rodent models [10], that there may be very early CNS involvement in HAT at a stage when patients are diagnostically classified as early stage and effectively treated with suramin. While further research is required into this phenomenon, one possibility is raised by observation that trypanosomes may readily penetrate the vascular endothelial basement membrane in some regions of the brain while still being unable to traverse the parenchymal basement membrane [34]. Such a process would bring trypanosomes in contact with astrocytes, and thus initiate neuroinflammatory responses. An increase in TNF-α concentration was only observed in late stage cases, and therefore is consistent with the mouse model of CNS infection [8]. With respect to the reduction in CSF TGF-β concentration with stage progression, while we were unable to measure CSF TGF-β in a sympatric control population, published data on CSF TGF-β levels in normal subjects (266 pg/ml [35]) suggest that in both early and late stage of infection TGF-β CSF concentrations are reduced below control levels. The reciprocal relationship of TGF-β and TNF-α concentrations in the CSF observed in disease progression is consistent with the mutually antagonistic anti- and pro-inflammatory properties of these two cytokines. TNF-α is a mediator of inflammatory neuropathology and its expression has been observed in association with astrocyte activation in mouse models of late stage HAT [36] as well as having been associated with disease severity in HAT [37]. TGF-β functions as a regulatory cytokine that can modulate inflammatory reactions in the CNS [38], for example through suppression of pro-inflammatory TNF-α expression in astrocytes [39], and the host TGF-β response has been implicated in the mild presentation of HAT observed in Malawi [23]. However, this does not account for the increase of CSF IL-10 with disease progression, as IL-10 is also an anti-inflammatory mediator [40]. The strong correlation between TNF-α and IL-10 levels in the CSF indicates the activation of distinct cellular compartments in the CNS during infection, and identification of the CNS cellular sources of IL-10, TNF-α and TGF-β in HAT will require further work in model systems, and to determine if distinct subsets of brain macrophages are involved in inflammatory/counterinflammatory regulation as has been observed in *in vitro* models of murine brain macrophage activation [41]. We observed no difference in CSF IFN-γ concentration between early and late stage. This result is not consistent with the close relationship of brain IFN-γ synthesis with disease progression described in the mouse model of African trypanosomiasis [8].

Cytokines in the CSF have previously been proposed as potential stage diagnostic markers [16], [19]. In this study, while IL-10 and TGF-β were predictive of diagnostic stage, they were insufficiently sensitive to be developed as effective staging markers, either individually or in combination with CSF IgM concentration.

Through analysis of immunoglobulin and cytokine responses in the CNS in relation to stage progression in *T.b.rhodesiense* HAT, it is possible to test the hypothesis that neurological dysfunctions observed in HAT are manifestations of inflammatory neuropathology. Neurological signs (gait abnormalities, tremors, incontinence, cranial nerve neuropathy, somnolence and mild coma) were equally probable to be observed in both early and late stage cases, and the only significant difference in incidence of neurological symptoms was for moderate coma which was never observed in early stage cases. This result is consistent with observations in a larger multi-centre study in Uganda [20]. While somnolence was equally probable in early and late stage cases, its incidence did increase with increasing reported duration of infection. We then analysed whether any relationship existed between CNS humoral and cytokine responses and neurological signs. Intrathecal Ig and CSF cytokine synthesis did not vary according to the presentation of gait ataxias, tremors, incontinence, facial nerve palsies, somnolence and mild coma. Therefore these neurological symptoms of HAT may have a non-immunological basis. However, individuals presenting with moderate coma presented elevated intrathecal IgG, IgA and IgM synthesis as well as significantly increased CSF IL-10 and IL-6 concentrations. In one respect this finding is to be expected, as of all the neurological signs that were investigated, moderate coma was only observed in diagnostic late stage cases. However, the moderate coma cases showed no significant increase in CSF IFN-γ, TNF-α or TGF-β in relation to mild or no coma cases. This suggests that inflammatory cytokine responses including TNF-α and IFN-γ do not increase with disease severity and this is not consistent with findings in experimental models and other clinical studies [10].

Finally, we observed a small number (3) of cases of HAT with CSF WBC concentration 6 and 20/µl. These cases were classified as late stage cases, however it was noted that these were the only 3 cases in the study where trypanosomes were not detected in the CSF even after double centrifugation. It is intriguing to speculate that these cases might present the first indication that, as is the case in *T.b.gambiense* infection, there may be an “intermediate” or “early-second stage” of infection [13], [15] in *T.b.rhodesiense* HAT. However such an interpretation would require the study of considerably more cases of this type, and while all three cases showed normal BBB function, 2 of the cases presented an intense intrathecal IgM synthesis (Fig. 1b) despite neither of these individuals showing any neurological signs.

In conclusion, in *T.b.rhodesiense* HAT, increasing levels of intrathecal immunoglobulin synthesis and CNS pro-inflammatory cytokine expression were associated with disease progression from early to late stage, although these were of limited diagnostic value. While intrathecal immunoglobulin synthesis was associated with the development of coma, it was not associated with any of the other typical neurological sequelae of HAT, which were also not related to CSF inflammatory or counter-inflammatory cytokine levels. Neuroinflammatory responses in correctly diagnosed early stage cases and cases with a similarity to the intermediate stage of *T.b.gambiense* HAT suggest there may be an early CNS involvement prior to the detectable invasion of the brain by the parasite and that effects on the CNS may be mediated indirectly by the parasite while it is still localised in the haemolymphatic system.

## Supporting Information

Figure S1 **Quotient diagrams for CSF IgG, IgA and IgM in HAT.** (a) early stage and (b) late stage HAT patients. Values above the upper discrimination line (Q_lim_, bold) indicate intrathecal synthesis, with the intrathecal fraction being indicated with reference to the dashed lines representing 20%, 40%, 60% and 80% of total CSF Ig. In panel (b) the cases marked with solid triangles represent the 3 individuals with CSF WBC between 6 and 20 cells/µl.
(TIF)

Checklist S1 **STROBE checklist.**
(DOC)
