# Supplementary figures and images for: Stage Progression and Neurological Symptoms in Trypanosoma brucei rhodesiense Sleeping Sickness: Role of the CNS Inflammatory Response
Source: PLoS Negl Trop Dis. 2012 Oct 25;6(10):e1857. doi: 10.1371/journal.pntd.0001857 (PMC3493381; doi:10.1371/journal.pntd.0001857)

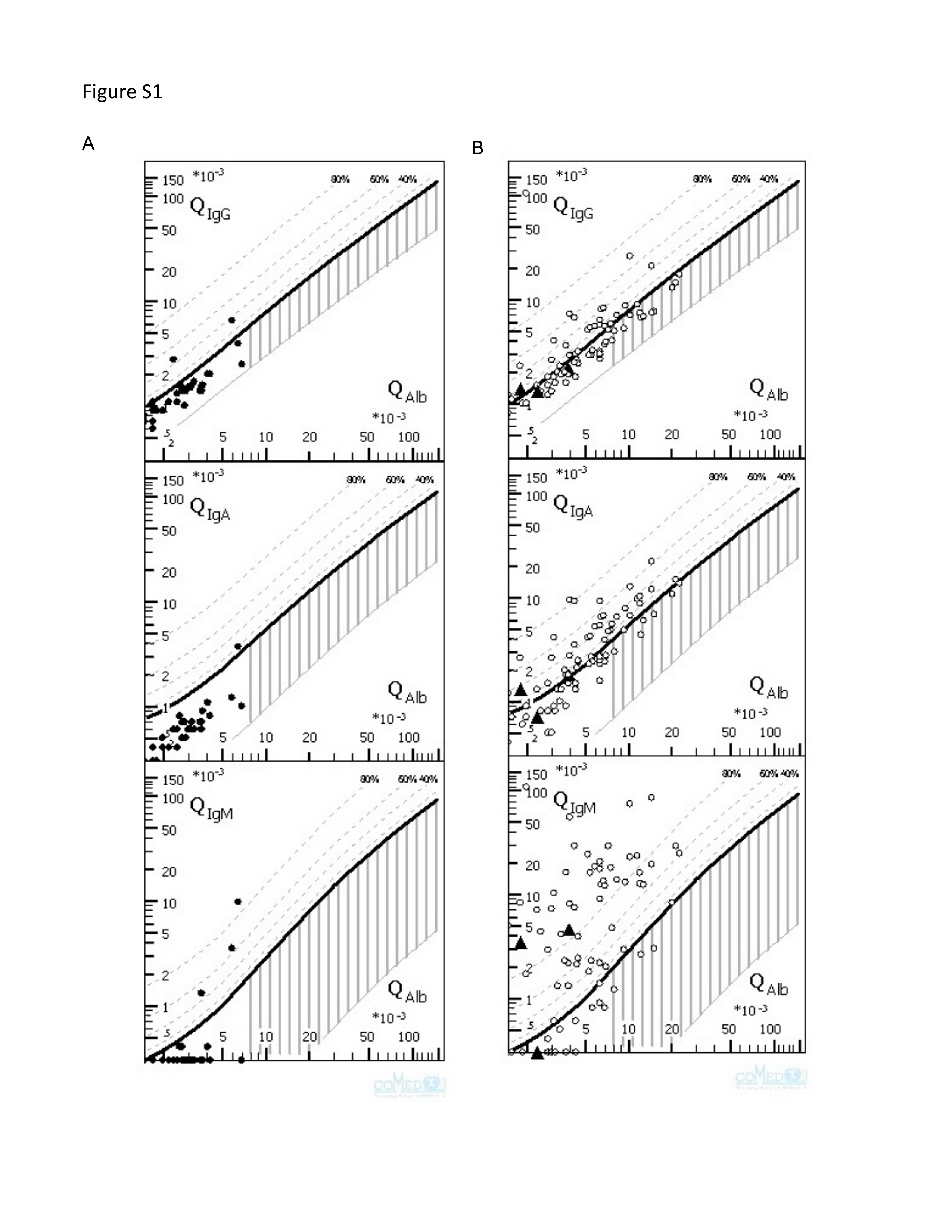

Supplement: Figure S1 — Quotient diagrams for CSF IgG, IgA and IgM in HAT. (a) early stage and (b) late stage HAT patients. Values above the upper discrimination line (Qlim, bold) indicate intrathecal synthesis, with the intrathecal fraction being indicated with reference to the dashed lines representing 20%, 40%, 60% and 80% of total CSF Ig. In panel (b) the cases marked with solid triangles represent the 3 individuals with CSF WBC between 6 and 20 cells/µl. (TIF) [file pntd.0001857.s001.tif]
